# Automating detection of diagnostic error of infectious diseases using machine learning

**DOI:** 10.1371/journal.pdig.0000528

**Published:** 2024-06-07

**Authors:** Kelly S. Peterson, Alec B. Chapman, Wathsala Widanagamaachchi, Jesse Sutton, Brennan Ochoa, Barbara E. Jones, Vanessa Stevens, David C. Classen, Makoto M. Jones

**Affiliations:** 1 Veterans Health Administration, Office of Analytics and Performance Integration, Washington D.C., District of Columbia, United States of America; 2 Department of Internal Medicine, Division of Epidemiology, University of Utah, Salt Lake City, Utah, United States of America; 3 Veterans Affairs Health Care System, Salt Lake City, Utah, United States of America; 4 Veterans Affairs Health Care System, Minneapolis, Minnesota, United States of America; 5 Rocky Mountain Infectious Diseases Specialists, Aurora, Colorado, United States of America; 6 Division of Pulmonary & Critical Care Medicine, University of Utah, Salt Lake City, Utah, United States of America; Drexel University, UNITED STATES

## Abstract

Diagnostic error, a cause of substantial morbidity and mortality, is largely discovered and evaluated through self-report and manual review, which is costly and not suitable to real-time intervention. Opportunities exist to leverage electronic health record data for automated detection of potential misdiagnosis, executed at scale and generalized across diseases. We propose a novel automated approach to identifying diagnostic divergence considering both diagnosis and risk of mortality. Our objective was to identify cases of emergency department infectious disease misdiagnoses by measuring the deviation between predicted diagnosis and documented diagnosis, weighted by mortality. Two machine learning models were trained for prediction of infectious disease and mortality using the first 24h of data. Charts were manually reviewed by clinicians to determine whether there could have been a more correct or timely diagnosis. The proposed approach was validated against manual reviews and compared using the Spearman rank correlation. We analyzed 6.5 million ED visits and over 700 million associated clinical features from over one hundred emergency departments. The testing set performances of the infectious disease (Macro F1 = 86.7, AUROC 90.6 to 94.7) and mortality model (Macro F1 = 97.6, AUROC 89.1 to 89.1) were in expected ranges. Human reviews and the proposed automated metric demonstrated positive correlations ranging from 0.231 to 0.358. The proposed approach for diagnostic deviation shows promise as a potential tool for clinicians to find diagnostic errors. Given the vast number of clinical features used in this analysis, further improvements likely need to either take greater account of data structure (what occurs before when) or involve natural language processing. Further work is needed to explain the potential reasons for divergence and to refine and validate the approach for implementation in real-world settings.

## Author summary

Identifying diagnostic error is challenging since it is often found only through review so time consuming not all patient data can be reviewed, let alone in a timely fashion to potentially prevent harm. In this work we address this gap by proposing machine learning methods which leverage millions of patient encounters. Since such methods could potentially be automated, they could scale to identify situations when the diagnosis may not be correct, timely, or when there may be a risk of death to the patient. This approach was validated by clinicians and shows promise for continued development. Future work will be needed to translate this work to protect patients and support clinicians.

## Introduction

Diagnostic errors are harmful to patients, traumatic for providers, and costly for healthcare systems. A recent study showed that infectious diseases are one of three major disease categories causing the majority of misdiagnosis-related harms [1]. It estimated 40,000 to 80,000 deaths in hospitals in the US related to misdiagnosis. Diagnostic error evaluation can be conducted with instruments such as Safer Dx [2,3]to identify areas of improvement; however, these instruments require manual case review from clinicians with expertise and are most efficient when applied to known or probable cases of error. Thus, while such instruments are useful, they are not scalable to large populations or ultimately amenable to providing rapid feedback at the point of care. Since current gaps cannot be addressed with audits and voluntary reporting systems alone, additional capabilities are needed.

There are different approaches to measuring diagnostic accuracy. SPADE uses large amounts of administrative and billing data to quantify diagnostic errors where diagnosis or treatment change indicate an outcome which may have been preventable had the diagnosis or treatment occurred more promptly [4].This statistical approach quantifies diagnostic errors on average but is difficult to interpret on the individual level, lacking traceability. Others have long used expert rules, which are challenging to maintain and scale to additional diseases or contexts [5].

One means of scaling up capabilities is through automation, including machine learning (ML) where models may be applied in large volume. Machine learning models have already been used to predict infectious disease. Examples include sepsis [6–9], pneumonia [10–12], upper respiratory infections (URI) [13], urinary tract infections (UTI) [14,15], and skin and soft tissue infections (SSTI) [16]. The difference between documented and predicted diagnosis can represent a kind of diagnostic divergence. Because diagnostic specificity is likely lower when the stakes are low, we also need to classify acuity. Several prior studies predict adverse outcomes such post-procedural 30-day mortality or adverse events [17–19].

We developed automated models using electronic health records to construct a flexible approach to detect diagnostic divergence which considers infectious disease diagnosis as well as risk of potential mortality. This approach was then validated by expert reviewers.

## Materials and methods

### Ethics statement

This project was reviewed by the VA Salt Lake City Research & Development Committee and the Institutional Review Board at the University of Utah, and a waiver of consent and authorization was granted (127273).

### Data

This study was performed using data from the Veterans Health Administration (VHA) Health Care System which cares for more than 9 million living Veterans at over one hundred emergency departments [20]. The study population included all emergency department (ED) visits to a VA medical center from January 1, 2017, to December 31, 2019. Data were extracted from the Corporate Data Warehouse (CDW), VHA’s repository for electronic clinical and administrative records.

Short visits with either little or no clinical detail (e.g., patients visiting ED for a medication refill) were excluded from further analysis if they did not have a minimum of 5 features in the first 24 hours of the ED visit. This minimum feature threshold was set to avoid uninformative visits. Additionally, ED visits were excluded from these sets if the patient was on hospice care or placed on hospice care within 72 hours. While both exclusions were made in consultation with a technical expert panel, the latter creates the possibility of missing diagnostic errors that lead to hospice and death. However, including these data in the training set would lead to learning hospice practices as “normal” and thus the decision was to exclude.

### Feature extraction

Features for each visit were extracted from clinical data starting at the time of the visit through 24 hours following the emergency department encounter, including inpatient admission for patients who were subsequently hospitalized.

Features for the present ED visit included orders, medications, laboratory results, radiology imaging results, and vital signs. Medical orders were normalized where possible to values in standard vocabularies. Specifically, when possible, these orders were Logical Observation Identifiers Names and Codes (LOINC) for laboratories, RxNorm for medications, and Current Procedural Terminology (CPT) for procedures [21–23]. Medication features were normalized to RxNorm ingredient level. Laboratory results standardized to LOINC were assigned to categories High, Low, or Normal based upon available structured data flags, categorical findings, or numerical results with respect to reference ranges (e.g., a serum creatinine of 2.5 mg/dL was simply coded a high serum creatinine). Radiology imaging results were categorized as either Normal or Abnormal based on an available structured data flag. Vital signs were only included as features if noted as abnormally High or Low.

To allow context of existing comorbidities prior to the visit and equal lookback, all diagnosis codes from the 365 days prior to the ED visit were extracted from both inpatient and outpatient settings.

All features were treated as binary, setting as true only if the specific event was documented for the patient during the encounter. As concrete examples, a feature name such as “order_comprehensive_metabolic_panel” would be present only if this order was present. The laboratory result was represented in a separate feature using suffixes added to feature names. For example, “lab_hemoglobin_n” represented that a laboratory for hemoglobin had a normal result. “lab_erythrocytes_l” and “lab_leukocytes_h” were interpreted similarly except that “l” and “h” represented Low and High results, respectively. Radiology imaging results such as “radiology_x_ray_exam_of_knee_a” and “radiology_x_ray_chest_left_view_n” represented Abnormal and Normal results, respectively. Vital sign example features of “vitals_blood_pressure_h” and “vitals_temperature_l” represented High and Low findings, respectively. Orders for medications were represented as “order_gabapentin” to reflect the order was made and a feature of “medication_gabapentin” if this medication was administered. Finally, historical diagnosis codes before the ED visit such as “Chronic obstructive pulmonary disease, unspecified” were represented as “icd10_dx_J44.9”.

### Data splits

Included ED visits were split into 3 datasets: training, validation, and testing. The testing set was defined to ensure that a set of ED visits were held out from all training and validation activities. [24]. Because documentation may vary over time within a medical center or across medical centers, the testing set was defined to include ED visits from facilities from July 1, 2019, to Dec 31, 2019. Additionally, five VA medical centers were selected at random for this set (including all their data for the entire time period). All remaining ED visits were assigned to the training and validation sets where 90% of visits were randomly assigned to the training set and the remainder to the validation set.

### Classification models

Two separate classification models were trained to predict outcomes in the proposed diagnosis metric. These are referred to here as the mortality and infectious disease models.

The mortality model was trained as a binary classifier where the label was defined by whether the patient died in the 30 days following the visit regardless of cause. Predictors included Elixhauser score using diagnosis codes from the 365 days prior to the visit [25] as well as other features mentioned above.

The infectious disease model was a multiclass classifier which predicted one of the following: pneumonia, sepsis, SSTI, UTI, URI, or no infection. These diagnosis outcome labels were assigned using sets of ICD-10 codes from previously published studies on pneumonia [26], sepsis [27–29], SSTI [30–32], UTI [33,34]. Labels for URI were assigned by manual curation of 45 ICD-10 codes for bronchitis (e.g., J20.9), pharyngitis (e.g., J03.91), sinusitis (e.g., J01.21), or generally for upper respiratory infection (e.g., J06.9). The definition for UTI also included microbiology results to expand the evidence used in assigning a label. A diagnosis was present for a visit if any one of these diagnosis codes (or microbiology data for UTI) was identified between in the period from 24 hours prior to 48 hours after an ED visit.

Both mortality and infectious disease models were trained using gradient boosted trees with the scikit-learn [35] package in Python. While many types of models could be trained and evaluated in this work, given available computing resources, one model type was selected for evaluation. Specifically, an implementation of gradient boosted trees, specifically XGBoost [36] was chosen for its familiarity and its proficiency in managing overfitting to promote generalization. Additionally, this implementation has been shown to scale to millions or billions of data instances while requiring fewer computing resources than some model types [36]. This implementation includes several hyperparameters which enforce model regularization to prevent models from becoming overly complex and fit to training data alone.

Hyperparameters for both models were tuned using randomized search and cross validation [37,38]. This was performed on the training set only using 3-fold cross validation and the best model with the best hyperparameters was measured against the validation set using the scikit-learn implementation. No metrics were gathered for the testing set until hyperparameters were finalized for both models. Some of the hyperparameters for this model included learning rate, maximum number of trees, and maximum depth permitted per tree. No explicit feature selection or feature weighting was performed prior to model training as each tree constructed in the training iterations could incorporate different features.

Negative classes were under sampled so that as many visits available could be leveraged while minimizing computational resource requirements. We used positive predictive value (PPV), sensitivity, and Area Under the Receiver Operating Characteristic (AUROC) to assess model performance. The area under the precision-recall curve (PRAUC) was also used as a metric since it has been shown to be informative in assessing performance of imbalanced classes [39].

### Diagnosis deviation

Using the predictions from the trained models as well as the diagnoses assigned and the estimated pre-visit mortality, a formula was developed to quantify potential deviation in diagnosis. This formula includes the predictions from the infectious disease classification model while also being weighted by predictions from the mortality model for increased mortality. This mathematical derivation of this diagnostic deviation (DD) is illustrated in the equation presented here.


$$DD=(d-\hat{d})*max\{(m-\hat{m}),0\}$$

More specifically, for any given infectious disease class (e.g., pneumonia) d represents the observed diagnosis, or a 0/1 representation of whether an ICD-10 code for that diagnosis, $\hat{d}$ represents the probability of that disease given predictions from the infectious disease classifier. Meanwhile, m reflects the pre-visit mortality probability from the Elixhauser score leading up to the visit when available and $\hat{m}$ represents the probability from the mortality model predicting 30-day mortality. Note that only increases in mortality probability are considered in this calculation so that if the probability of mortality during the visit decreases compared to the pre-visit mortality, this term will be 0.

### Validation

To validate how well this proposed automated metric compares to clinician judgement, ED visits were manually reviewed. Reviewers assessed whether each of the five infectious disease classes or a non-infectious disease was initially documented. Next, changes in diagnostic approach or the investigation of multiple diagnoses were assessed, and for the same disease classes, identified the final diagnosis for the reason the patient came to the ED.

Additionally, two questions were adapted from the revised Safer Dx framework to rate opportunities for improved diagnosis [3]. On a Likert scale of 1 (i.e., strongly disagree) to 7 (i.e., strongly agree), two questions were asked: “*The final diagnosis was not an evolution of the care team’s initial presumed diagnosis*” and “*The patient was at risk significant harm–or experienced harm–that could have generally been prevented by a correct and timely diagnosis*”.

Case reviews were performed by 3 clinical experts in infectious disease. After an initial exploratory round of chart reviews, the scope of the reviews was narrowed to pneumonia given available time for reviewers. Cases assigned to each reviewer were a stratified sample where half were assigned by the highest values according to our metric with respect to pneumonia and the other half were randomly sampled. Cases were excluded from sampling if the difference between expected mortality (m) and predicted mortality ($\hat{m}$) reflected a decrease.

The three reviewers triple annotated a set of 20 ED visits to measure inter-rater reliability (IRR) between reviewers. After these visits, reviewers were asked to continue reviewing visits as they had available time such that a total of 130 unique ED visits were reviewed. After reviews were completed, we assessed the correlation between the diagnosis deviation and the Likert scores for each of the two questions using weighted Cohen’s kappa [40]. We also used Cohen’s kappa to evaluate IRR on the cases reviewed by multiple reviewers.

Comparisons between diagnosis deviation and reviewer scores on the two questions for review were calculated by Spearman rank correlation [41].

## Results

### Data

A total of 6,536,315 ED visits were initially included from 104 distinct VA medical centers. These visits were across 2,141,271 unique patients where the mean age at the time of the ED visit was 60 years old and 88.1% were male.

### Feature extraction

From these ED visits, over 738 million features were extracted. Over 656 million features were extracted with respect to the first 24 hours of data in the ED visit and over 82 million features were extracted from diagnosis codes prior to the visit. A summary of feature counts and distinct feature values is shown in Table 1. The median number of features in the first 24 hours of the visit was 22 and the median number of diagnosis codes prior to the visit was 8.

**Table 1:** Counts of features for each type as well as the count of distinct features values in each.

Feature type	Total feature count	Distinct feature values
Lab results	265,214,795	8,338
Medication	23,391,304	348
Radiology results	7,171,729	1,367
Orders	239,030,254	10,309
Vital signs	121,842,454	7
Outpatient diagnosis codes	82,011,683	39,770
Inpatient diagnosis codes	186,022	9,408
All features	738,848,241	69,547

### Data splits

A total of 4,261,730 visits were divided into sets. 1,211,698 visits were assigned to the testing set where 641,765 were from the temporal holdout and 569,933 were from one of the five random medical centers. The remaining visits were split among the training set and validation sets which resulted in sets of 2,733,493 and 316,539 visits, respectively.

### Classification models

Following hyperparameter tuning and cross validation of both the mortality and infectious disease models using the training and validation sets, these models were finalized and applied to the held-out testing set.

**Fig 1:**
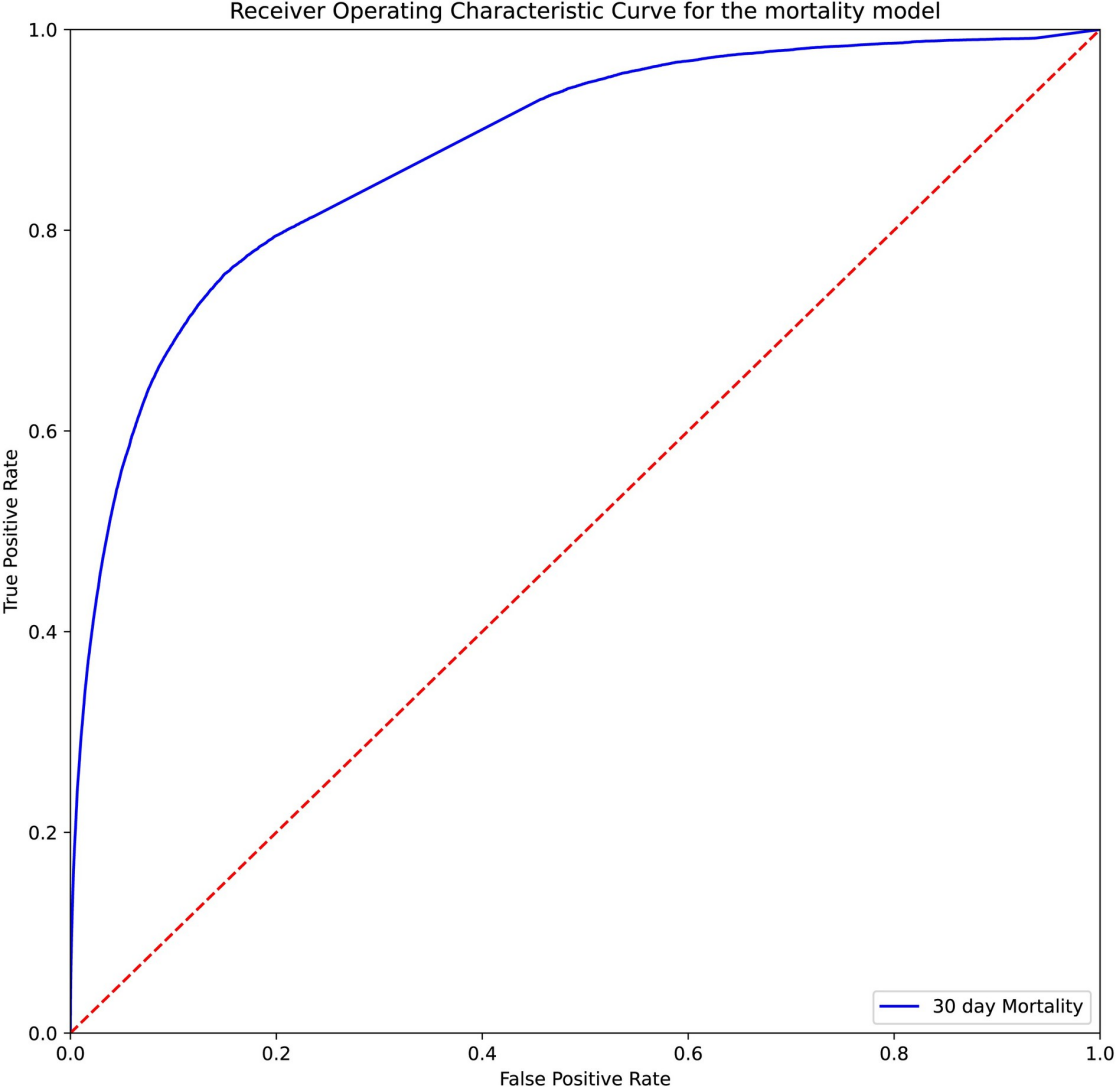
Receiver operating characteristic curve for the mortality binary model on the testing set.

**Fig 2:**
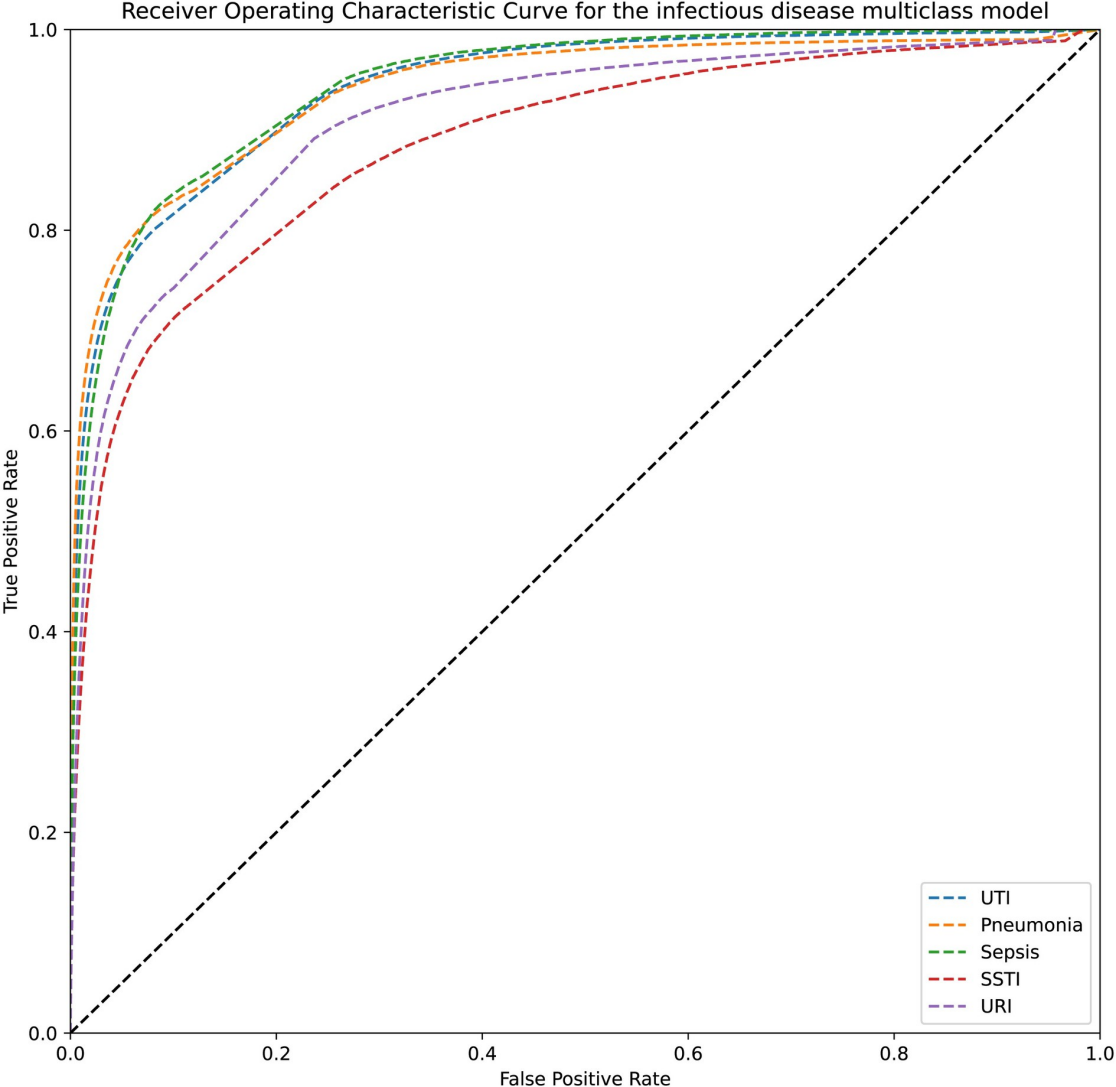
Receiver operating characteristic curve for the infectious disease multiclass model on the testing set.

Performance metrics on this testing set for the mortality model is shown in Table 2. The metrics for the same set for the infectious disease model is in Table 3. The receiver operating characteristic curves for these models on the testing set are shown in Fig 1 and Fig 2, respectively.

**Table 2:** Performance of the mortality binary model on the testing set.

	PPV	Sensitivity	F1	AUROC	PR AUC	Count
No Mortality	98.3	99.9	99.1	89.1	99.7	1,189,602
Mortality	61.0	9.0	15.7	89.1	26.3	22,096
Macro average	97.7	98.3	97.6	-	-	1,211,698

**Table 3:** Performance of the infectious disease multiclass model on the testing set.

	PPV	Sensitivity	F1	AUROC	PR AUC	Count
No infection	90.0	96.2	93.0	90.6	97.2	970,054
Pneumonia	77.6	53.1	63.1	94.7	66.8	43,573
Sepsis	50.7	30.9	38.4	93.8	37.8	26,657
SSTI	53.7	28.6	37.3	91.9	39.8	12,917
UTI	78.3	61.6	69.0	94.3	74.1	94,578
URI	64.8	49.3	56.1	92.2	56.2	63,919
Macro average	86.1	87.2	86.7	-	-	1,211,698

### Diagnostic deviation

**Fig 3:**
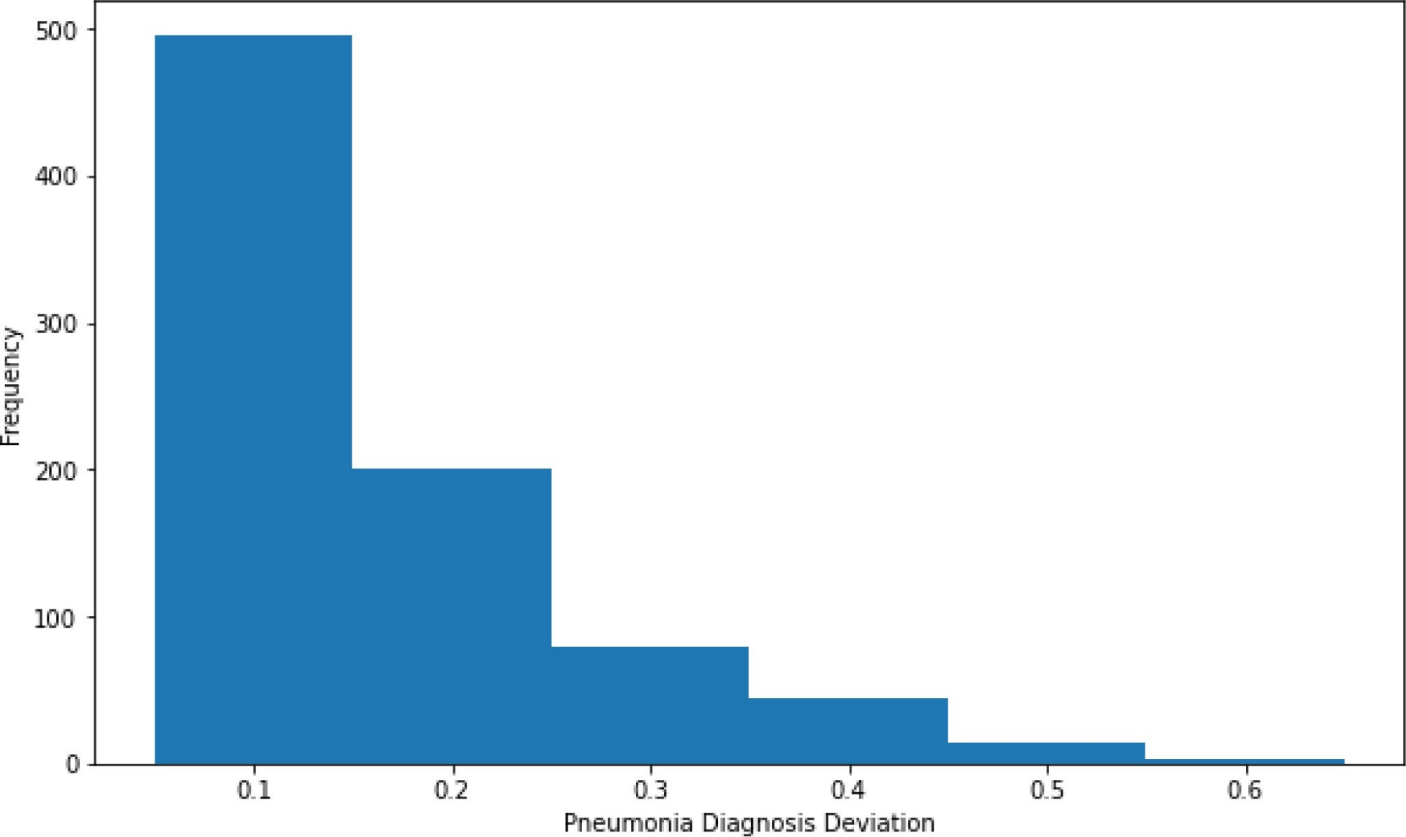
Distribution of the Diagnostic Deviation for Pneumonia for each visit in the test set of this analysis. Since most values are 0, this is not shown.

After models were trained and validated, the proposed Diagnostic Deviation Metric was applied to all ED visits in the testing set. The distribution of this metric for pneumonia is shown in Fig 3.

### Validation

**Fig 4:**
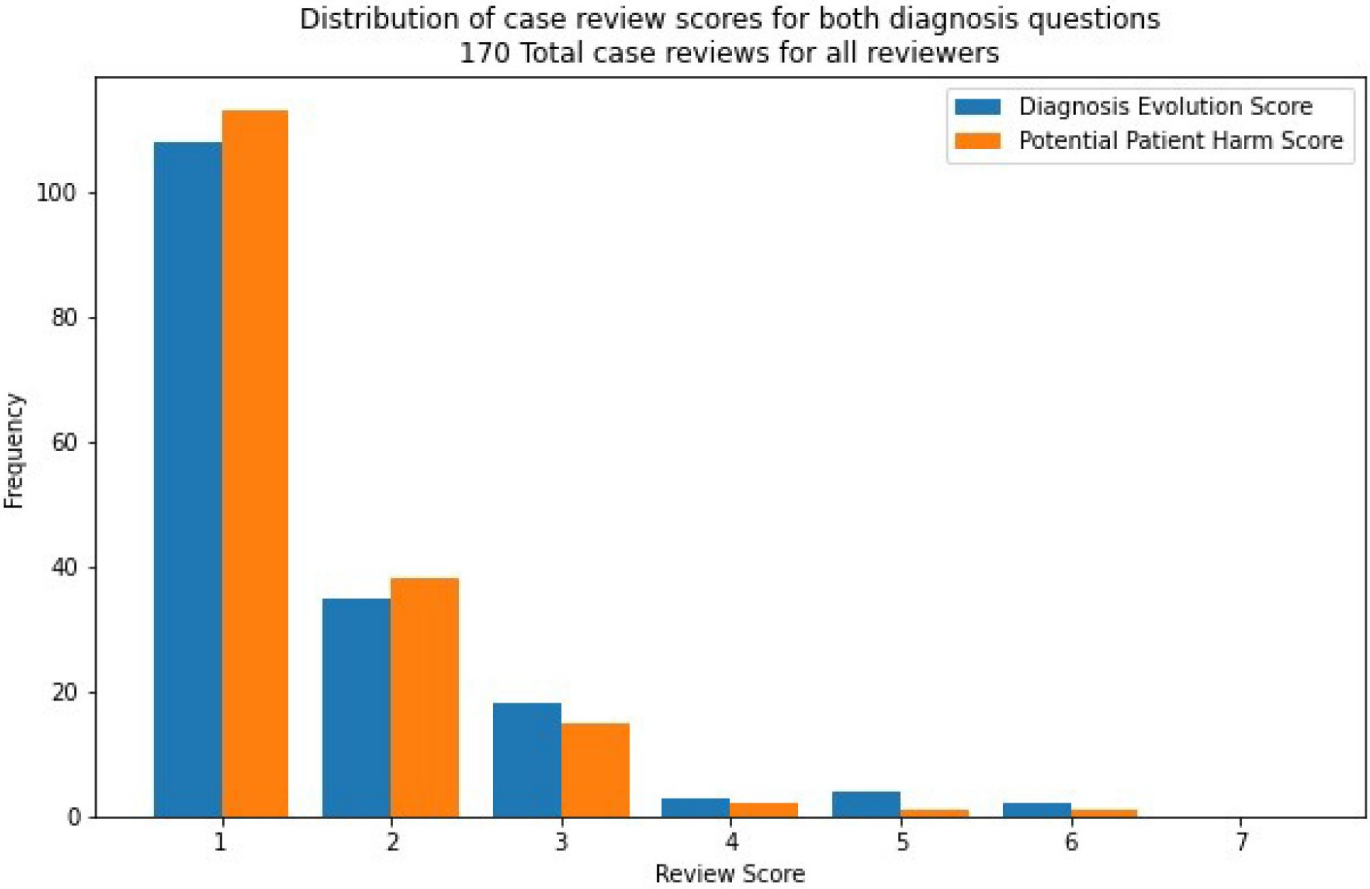
Distribution of all scores assigned by all three reviewers for the two questions asked on each reviewed case.

The distribution of values assigned during review is shown in Fig 4. Pairwise inter-rater reliability is shown in Table 4 where the Cohen’s Kappa ranges from 0.242 to 0.528 across both questions posed.

**Table 4:** Inter-rater reliability between pairs of reviewers. Each question is measured using weighted Cohen’s Kappa.

Comparison for IRR	Not a diagnostic evolution	Potential patient harm
Reviewer 1 vs 2	0.242	0.419
Reviewer 1 vs 3	0.319	0.377
Reviewer 2 vs 3	0.528	0.364

Since our proposed measure combines predictions of both mortality and diagnosis, human review scores were not only compared to the composite metric, but also to each of these components of the measure independently. These correlations for all reviewers are presented in Table 5. Our proposed composite measure shows a positive correlation between human reviewers and this automated approach which may be interpreted as weak to moderate given the Spearman correlations between 0.231 and 0.358. The composite metric also showed a higher correlation than the individual components of mortality or diagnosis deviation, although the difference was greater in the human score of diagnostic evolution than it was for potential patient harm. A correlation for each reviewer among the two questions response is provided in Table 6. For the question of “not a diagnostic evolution,” correlations ranged from 0.35 to 0.614 with Reviewer 1 showing the strongest correlation. Meanwhile, for potential patient harm, these ranged from 0.273 to 0.357 where the strongest correlation was with Reviewer 2.

**Table 5:** Comparisons of the individual components of our metric as well as the composite metric when compared to each of the human review scores for all reviewers.

Metric (or component) for comparison	Human Review Score	Correlation
Mortality deviation only	Potential patient harm	0.231
Diagnosis deviation only	Not a diagnostic evolution	0.317
Composite metric	Potential patient harm	0.247
Composite metric	Not a diagnostic evolution	0.358

**Table 6:** Correlation between our proposed metric (DD) and each reviewer by responses to each question. Each question is measured using Spearman rank correlation.

Reviewer	Cases Reviewed	Correlation of “Not a diagnostic evolution” review and DD measure	Correlation of “Potential patient harm” review and DD measure
Reviewer 1	100	0.354	0.273
Reviewer 2	50	0.350	0.357
Reviewer 3	20	0.614	0.311

Reviewers were allowed to provide comments to summarize cases including whether they agreed with the diagnoses assigned in the cases, noteworthy outcomes in the cases and any other useful notes. Some review scores had higher correlation with our proposed measure and comments provided by the reviewer seem to indicate that an automated review may have been useful. Some of these comments are presented in Table 7. Some of these include cases where one treatment was provided, but pneumonia was missed as the diagnosis. Others indicate an initial diagnosis, but by the time the diagnosis changed there was an adverse outcome.

**Table 7:** Comments from reviewers on case reviews where the measure was helpful and correlated with reviewer scoring.

Reviewer Comments
Originally diagnosed as pneumonia or septic shock but was actually non-ST-elevation myocardial infarction (NSTEMI).
Treated for liver failure but missed pneumonia.
Treated as urinary tract infection (UTI), eventually pneumonia was raised as a diagnosis, but the patient died.
Thought to be septic shock, patient later had an acute myocardial infarction (AMI).

Given that the correlation between the proposed measure and review scores is suboptimal, there were also cases where the score from the measure may have been high, but the reviewer did not identify an opportunity for a more timely or correct diagnosis. These are shown in Table 8. For example, in some of these the reviewer agreed with the diagnoses assigned in the cases. In at least one of these, a reviewer indicated that the patient was already in hospice, which should have been excluded from our analysis and review.

**Table 8:** Comments from reviewers where the measure was not clearly helpful and not correlated with reviewer scoring.

Reviewer Comments
Treated in ED for GI issues; Pneumonia likely new and not present at admission
Complex case. Already existing pneumonia. At the end of the course, considered non-infectious causes for findings.
The team probably got the right diagnosis of COPD exacerbation.
Patient already on hospice, then pneumonia and NSTEMI.

## Discussion

Individual models for infectious diseases and mortality demonstrated reasonable diagnostic performance statistics but positive predictive value and PR AUC were low given the low prevalence of infectious diseases. These models were trained with a very large number of features, representing a substantial fraction of the structured data readily available from an EHR.

Our primary concern was whether our combined measures of diagnostic divergence was related to diagnostic error. Correlations between human review of cases and the proposed measure showed a weak positive correlation. When examining the interrater reliability of our subjective diagnostic error measures and when performing an analysis of discordant cases, it became apparent that there was substantial disagreement in interpreting cases and how to weigh the individual components of diagnostic error. This suggested a further need to explore robust instruments for diagnostic error assessment. It also underscores that diagnostic error is usually not clearly present or absent but is evaluated on a continuum. Analysis also showed that cases with high diagnostic divergence scores that were obviously not diagnostic errors generally fell into three categories: diagnostic miscoding, highly complex cases, and model failures, of which the first two were most common.

By searching on the very high end of the diagnostic divergence spectrum, we demonstrated the feasibility of enriching data sets with infectious disease-related diagnostic error cases using automated means. Such identified divergence could be used to highlight a diagnosis that should have been included or to offer information where the assigned diagnosis differs from how peers may have assigned a diagnosis. Further, leveraging methods for explainable machine learning could help to identify which specifics of diagnostic testing or treatment contributed to this divergence.

The performance of our individual models was similar to other reported ML models to date when comparing common metric like AUROC, although study criteria and settings make comparisons non-trivial [6–8,11,12,14–19]. However, given the amount of data and number of features, this is somewhat disappointing. We included millions of clinical events in modeling efforts in which thousands of model iterations were trained and evaluated with a range of hyperparameters. This suggests that the features added and that are not usually present in other models did not provide much additional information over more traditional features when predicting the ultimate diagnosis. This may be, in part, due to sparse documentation on observations supporting the diagnosis and/or selective documentation of features supporting the diagnosis, which would make it difficult to identify diagnostic error as well. This work has limitations, particularly with respect to assumptions made with the data. First, since ICD-10-CM codes are used in training and validation of the infectious disease classification model, there are both false positives and false negatives in these assigned codes with respect to the actual working diagnosis, [42–44], e.g., a provider may diagnose dyspnea (a non-specific diagnosis) but be thinking about and treating pneumonia correctly. Issues like these are not unique to infectious disease, but there may be opportunities to improve classification by intent via other data sources such as medications, results from laboratory or microbiology specimens, or from text processing. The inter-rater reliability of our instruments for assessing diagnostic error was low, so it is difficult to interpret correlation with our diagnostic divergence score. Also, further work on ontologies could help to disambiguate when the documented code represents an error as opposed to simply a different stage along a natural evolution of the diagnostic workup.

We also categorized quantitative results (e.g., laboratory values) which could have destroyed predictive information. We did not use unstructured data which could have provided more nuance. Models did not consider temporal sequence which also could have lost predictive information. However, even with this feature engineering, we still had a very large number of features. It may be necessary to roll-up features for optimal learning, perhaps with the use of ontologies [45]. An additional limitation is that while many model types exist, this work selected one type of model and evaluated it exclusively. In continued work, it would be worthwhile to evaluate other types of models to compare their performance on these tasks, as well as their respective computational burdens.

Another limitation is that while this VA data comes from several medical centers with diverse geographic locations, our findings and methods may not generalize to other healthcare systems; however, we believe that the same approach of model training and divergence measurement will hold. In this study, patients placed on hospice care were excluded, thereby excluding an important population for whom errors may have occurred. We believe that this is likely unavoidable without precise documentation of when hospice begins to be seriously considered. Training on data of patients on hospice without a diagnostic workup may result in models learning that this pattern is not as unusual as it should be outside of the hospice setting.

Perhaps most importantly, we were limited by a lack of a gold standard—hampered both by data to train models on and a large data set to validate against. The first problem prompted our use of anomaly detection methods in the first place and will require further research. The second underscores a lingering lack of conceptual clarity around a multifaceted concept that also merits further work.

While performance of these models may be acceptable for initial feasibility, future studies should optimize for other data sources and methods to improve model performance as both PPV and sensitivity are relatively low on some classification classes. We also aim to develop methods to provide better interpretation of flagged cases. Several studies with clinicians have shown that merely showing a score is not enough—clinicians benefit from more rationale for why a model made its prediction and what action might be taken with the information [46–48].

We imagine that this approach could eventually be used to support quality-oriented chart reviews by retrieving records enriched for diagnostic error. To be routinely implemented, however, the positive predictive value will need to be improved. Before use in other healthcare systems, issues of generalizability will also need to be established in other environments. Evaluation methods also need to be improved to more adequately refine the types of misdiagnoses that we hope to focus on. We expect that besides continued improvements in the adaptation of anomaly detection algorithms to detect misdiagnosis, that normal data (through improved data quality, reduced miscoding, and documentation) will cluster together more clearly thereby making anomaly detection of misdiagnosis easier to detect. In the future, we anticipate that this approach will be accurate enough in detecting misdiagnosis that it could be incorporated into quality and safety measures.

## Conclusion

Our proposed method for detecting diagnostic deviance yields candidate cases enriched for diagnostic error. It also finds miscodes and difficult cases. Further refinement could yield a tool for flagging charts for review. Comparisons between human review and our approach indicate preliminary feasibility. Increases in accuracy will likely require natural language processing and methods to leverage information on time and concept relatedness. Further work is needed to develop reliable instruments for rapidly evaluating diagnostic error. Continued development is necessary to allow reviewers and users to explore more detailed information and be convinced that the measurements are valid before a metric can be implemented in clinical practice.
